# A case report of venous air embolism induced by bone marrow cavity irrigation

**DOI:** 10.1097/MD.0000000000046455

**Published:** 2026-01-16

**Authors:** Boe Feng, Hong Duan, Kai Liu, Li-ming Cheng

**Affiliations:** a Department of Anesthesiology, Kunming Children’s Hospital & Children’s Hospital of Kunming Medical University, Kunming, China; b Comprehensive Pediatrics, Kunming Children’s Hospital & Children’s Hospital of Kunming Medical University, Kunming, China.

**Keywords:** bone marrow cavity irrigation, echocardiography, emergency management, pediatric surgery, venous air embolism

## Abstract

Venous air embolism (VAE) is a rare but severe complication that can occur during surgical procedures that involve the exposure of vascular structures or pressurized irrigation. We present a rare instance of severe VAE in a child that transpired during the irrigation of the bone marrow cavity during surgery for femoral osteomyelitis in a 3-year-old girl. The patient experienced a rapid clinical deterioration shortly after the commencement of irrigation with diluted povidone-iodine and saline. This deterioration was characterized by a sudden decrease in end-tidal carbon dioxide, oxygen saturation, pulse rate, and blood pressure. A bedside echocardiogram promptly confirmed the presence of intravascular air bubbles. Rapid symptom relief was achieved through prompt emergency interventions, which included catheter aspiration, 100% oxygen, left lateral positioning, and irrigation cessation. The patient underwent a full recovery without any neurological sequelae. In pediatric orthopedic surgery, the significance of vigilance, immediate echocardiographic assessment, and timely treatment to prevent morbidity and mortality due to VAE is underscored by this case.

## 1. Introduction

Venous air embolism (VAE) is a potentially fatal event that results from the entrance of air into the venous circulation, which in turn causes hemodynamic collapse and obstruction of pulmonary blood flow.^[1]^ VAE is frequently found in neurosurgical and cardiovascular procedures; however, it can also occur during orthopedic surgery that involves exposed venous access or pressurized irrigation systems.^[2]^ The high physiological susceptibility to intravascular air and the small circulatory volume of pediatric patients render them particularly vulnerable.^[3]^ Even though VAE is uncommon in minors, it is frequently underestimated during surgery and poses a substantial risk.^[4]^ The presence of early symptoms, such as hypotension, hypoxemia, and an abrupt decrease in end-tidal carbon dioxide (ET CO_2_), should induce concern.^[5]^ Timely diagnosis necessitates point-of-care echocardiography, which allows real-time visualization of intravascular air and guides urgent intervention.^[6]^ Prompt diagnosis and intervention are essential due to the severe and swift nature of VAE. This case emphasizes the necessity of vigilance during the operation and underscores the occurrence of VAE during the lavage of the bone marrow cavity in pediatric patients.

## 2. Case statement

On December 20, 2023, the patient, a 3-year-4-month-old girl, was confined to the hospital for 4 days due to pain and edema in the left thigh, which was accompanied by a high fever for 1 day. The physical examination revealed edema in the left thigh, elevated local skin temperature, tenderness, and percussion pain that did not fluctuate. Elevated inflammatory markers (white blood cell count: 10.27 × 10^9^/L, CRP: 132 mg/L, ESR: 114 mm/h, and PCT: 1.65 ng/mL), hypoalbuminemia (23 g/L), anemia (hemoglobin: 83 g/L), and a positive MSSA blood culture were detected in laboratory assays. Osteomyelitis with abscess formation was indicated by magnetic resonance imaging. The patient’s clinical status markedly improved following the initial drainage surgery on December 20, and there were no signs of sepsis before the second procedure on January 2.

Due to recurrent femoral osteomyelitis and periosteal abscess, the patient underwent a second operation at Kunming Children’s Hospital, performed in the right lateral decubitus position for optimal left femoral exposure. Throughout the preoperative period and anesthetic induction, vital signs – including ET CO_2_ and hemodynamics – remained stable until the intraoperative event. Immediately following surgical debridement, the surgeon initiated the process of flushing the femoral medullary cavity with diluted povidone-iodine and saline using a syringe under mild pressure during the operation.

The patient experienced an abrupt and profound cardiopulmonary deterioration approximately 2 minutes after the initiation of medullary cavity drainage. The ET CO_2_ level declined rapidly from 35 to 10 mm Hg, and the noninvasive blood pressure decreased significantly from 110/70 to 62/38 mm Hg. Concurrently, her oxygen saturation decreased from 99% to 80%, and her heart rate decreased from 120 to 45 pulses per minute. Despite the rapid hemodynamic collapse, a palpable but feeble carotid pulse persisted, suggesting that central perfusion was temporarily preserved. The electrocardiogram demonstrated that the ST segments were depressed, which indicates myocardial ischemia.

**Figure 1.**
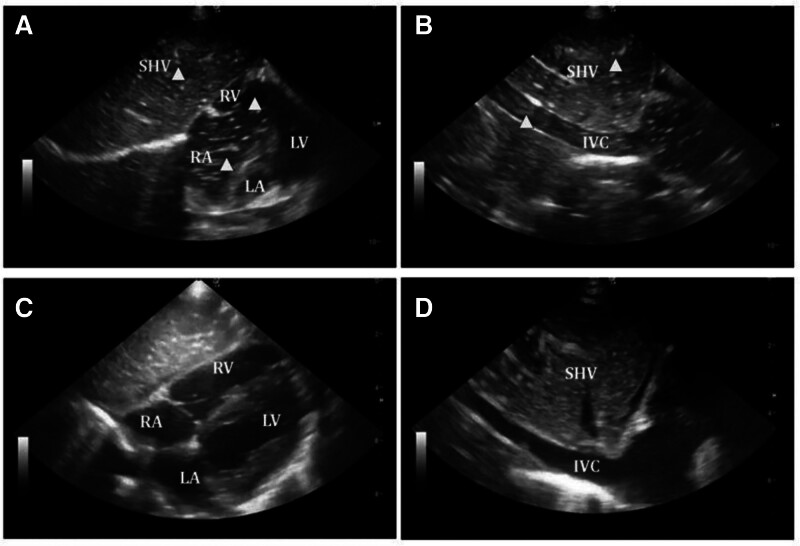
Subcostal echocardiographic views demonstrating VAE before and after emergency intervention. (A) Subcostal 4-chamber view shows multiple echogenic bubbles (▲) in the RA and RV before intervention. (B) Subcostal longitudinal view of the IVC reveals scattered intravascular air bubbles (▲) near the SHV. (C) After intervention, air bubbles are no longer visible in the RA and RV, indicating resolution. (D) The IVC is clear of bubbles posttreatment. ▲ = intravascular air bubbles, IVC = inferior vena cava, LA = left atrium, LV = left ventricle, RA = right atrium, RV = right ventricle, SHV = subhepatic vein, VAE = venous air embolism.

Other differential diagnoses, including anaphylaxis and fat embolism syndrome, were promptly considered and ruled out based on the clinical features, specifically the absence of rash, bronchospasm, petechiae, neurological deficits, or characteristic radiographic findings. The abrupt cardiopulmonary deterioration, which was closely associated with intramedullary irrigation, induced a strong suspicion of VAE. The diagnosis was confirmed by the immediate performance of a point-of-care echocardiogram, which revealed numerous air pockets in the right atrium (RA) and inferior vena cava (IVC) (Fig. 1A and B).

Emergency interventions were implemented without delay. The surgical team discontinued irrigation and draped the surgical field with saline-soaked gauze to prevent additional air from entering. The patient was positioned in a steep Trendelenburg and left lateral decubitus position to alleviate pulmonary outflow obstruction and facilitate air entrapment at the apex of the RA. Simultaneously, 100% oxygen was administered through mechanical ventilation to reduce nitrogen discharge and expedite the resorption of intravascular air. A 4F double-lumen central venous catheter was promptly inserted into the right internal jugular vein and connected to negative pressure aspiration under ultrasound guidance to evacuate intracardiac air.

The bone marrow cavity irrigation was performed manually using a 20 mLsyringe, applying gentle manual pressure estimated at 100 to 150 mm Hg.

It is estimated that 15 mL of air was aspirated from the central catheter in multiple draws, with an average of 2 to 3 mL of air per 10 mL of blood withdrawn, repeated 6 times. This procedure substantially reduced the amount of intravascular air visible on echocardiography. Epinephrine infusion via central venous catheter was initiated to rapidly stabilize blood pressure and maintain adequate cardiac output.

The patient’s hemodynamic condition progressively improved over the next 10 minutes. The volume of intravascular air was significantly reduced, as evidenced by repeat point-of-care echocardiography (Fig. 1C and D). She was transferred to the surgical intensive care unit for continuous monitoring, where she was sedated and maintained on mechanical ventilation. No symptoms of delayed complications or embolic sequelae were observed during her recovery, and her neurological function remained unaffected. The head magnetic resonance imaging and thoracic computed tomography scans were unremarkable, except for cerebral or pulmonary injury. The patient was effectively extubated on the second postoperative day and was discharged from the intensive care unit on the 4th day. She was alert, hemodynamically stable, and neurologically normal by the fifth postoperative day (Glasgow Coma Scale score of 15). On the 14th postoperative day, she was discharged home in excellent general condition, with normal respiratory function and no residual deficits, after additional inpatient observation and supportive care.

This case demonstrates the significance of early recognition and rapid intervention in preventing morbidity and mortality associated with VAE in pediatric patients, as uncommon but severe complications can occur during what appears to be a routine procedure.

## 3. Discussion

VAE is an uncommon intraoperative complication that is susceptible to fatality. It is more prevalent in pediatric patients undergoing procedures involving open venous structures or pressurized irrigation exposure. Although most cases of VAE occur during neurosurgery, cardiovascular surgery, or laparoscopic surgery, the incidence of VAE in orthopedic surgery has not been wholly reported. Still, its clinical significance should not be overlooked.^[7]^ Indeed, in this instance, a 3-year-old girl experienced a severe and rapid VAE during the drainage of the femoral marrow. The clinical response and recovery demonstrate the importance of early detection, appropriate diagnostic instruments, and timely management in accordance with protocols.^[8]^

The occurrence of pediatric orthopedic-related VAE has been previously reported, albeit infrequently, during invasive bone procedures. A review of orthopedic procedures highlighted the often underestimated yet critical risk of VAE, underscoring the need for early recognition and prompt intervention. Our case emphasizes the significance of immediate intraoperative diagnosis and intervention, as it uniquely illustrates bone marrow irrigation-induced VAE with rapid bedside echocardiographic confirmation and successful multidisciplinary management, in contrast to their findings.^[9]^

VAE is the process by which air enters the venous circulation through an open or damaged vein under negative or low pressure and subsequently enters the right ventricle and pulmonary vasculature. In minors, even minute quantities of air can result in severe cardiopulmonary damage as a result of diminished compensatory reserves and reduced circulating blood volume.^[10]^ Ventilation-perfusion mismatching and severe hypoxia result from the air in the RA, which obstructs pulmonary arterial flow, reduces cardiac output, and impedes venous return.^[11]^

The earliest and most reliable clinical indicator of VAE is a sudden and abrupt decrease in ET CO_2_, which is indicative of the reduction in pulmonary blood flow resulting from air blocking the pulmonary vasculature.^[12]^ The suspicion of VAE was accurately indicated by the fact that ET CO_2_ decreased from 35 to 10 mm Hg within a few minutes. Hypoxaemia, hypotension, bradycardia, and ST-segment depression on the electrocardiogram are additional symptoms that are consistent with the pathophysiological cascade of acute VAE.^[13]^

The preferred diagnostic method for suspected intraoperative VAE remains point-of-care echocardiography. It can display air in the RA and IVC in real-time, enabling clinicians to verify the diagnosis and promptly monitor the efficacy of treatment.^[14]^ Transesophageal echocardiography is more sensitive but frequently less feasible in emergencies or pediatrics. In our case, the subxiphoid view of the RA and IVC plainly demonstrated the presence of air bubbles, which confirmed the diagnosis and guided subsequent treatment.

Numerous established principles manage VAE. A systematic review has comprehensively outlined the diagnostic criteria and management strategies for intraoperative VAE, reinforcing the effectiveness of immediate interventions such as oxygen administration, patient positioning, and aspiration via central venous catheterization.^[15]^

The initial stage is to obstruct the air entry point, which is achieved by discontinuing irrigation. To prevent further air entry, a temporary seal can be established by covering the surgical area with gauze that has been saturated in saline. In the interim, it is essential to administer 100% oxygen to increase oxygenation and facilitate the exhalation of nitrogen from the bubbles, thereby enhancing its absorption. The Durant maneuver, which involves positioning the patient in a left lateral decubitus position with the head lower than the feet, is designed to reduce the risk of further pulmonary artery embolization by confining air in the RA.^[16]^

In severe cases, aspiration through a central venous catheter may be lifesaving, mainly if echocardiography confirms the presence of a substantial quantity of air in the RA.^[17]^ This combination of interventions in our patient led to a rapid resolution of hemodynamic instability, with subsequent echocardiography verifying the removal of intravascular air.

Additionally, this instance underscores a critical prevention concern. Although irrigation is customary during bone surgery, using pressurized systems may result in danger if veins are exposed or injured. Clinicians should exercise caution when irrigating bone cavities, particularly in pediatric patients. Practical measures to mitigate the risk of VAE include maintaining the surgical field damp, preventing excessive pressurization, and ensuring adequate hemostasis.^[18]^

It is imperative to maintain normotension and euvolemia to reduce the risk of VAE by substantially diminishing negative venous pressures. Additionally, surgical fields should be positioned at a lower level than the heart whenever feasible, and pressurized infusion systems must include air-fluid level monitoring and alarm functionality. It is essential to reduce the amount of inspiratory effort to prevent the risk of venous air aspiration and the elevation of negative thoracic pressures during spontaneous ventilation.^[19]^

Given the vulnerability of pediatric patients due to open venous channels in bone marrow, irrigation under high pressure poses a significant risk for VAE. Adopting lower pressure, controlled manual irrigation techniques, as demonstrated in this case, is crucial for minimizing such risks.^[20]^

Furthermore, it is imperative to establish an emergency plan for VAE. This plan encompasses routine echocardiography, continuous monitoring of ET CO_2_, trained personnel capable of performing the Durant maneuver, and immediate access to a central venous access device for evacuation if necessary. Additionally, it is crucial to provide surgical and anesthesia teams with information regarding the signs, risk factors, and management strategies of VAE.^[21,22]^

This case illustrates that VAE can have severe repercussions in nontraditional high-risk procedures, such as pediatric orthopedic surgery. Rapid echocardiographic confirmation, early recognition through ET CO_2_ monitoring, and standardized management (including the cessation of the gas source, hyperoxia, positioning, and aspiration) are essential for patient survival and recovery. Despite the rarity of this condition, clinicians must exercise caution during comparable procedures to mitigate the likelihood of delayed recognition and adverse outcomes.^[23,24]^

This report describes a single pediatric case of VAE during femoral medullary irrigation, which inherently limits the generalizability of its findings. Hemodynamic and respiratory parameters were not continuously recorded in detail during the peri-event period, restricting the ability to fully characterize the physiological changes. The diagnosis relied primarily on point-of-care echocardiography without concurrent confirmation by other imaging modalities, such as transesophageal echocardiography or computed tomography. Furthermore, the irrigation pressure was estimated rather than directly measured, making it difficult to quantify the exact threshold associated with the event. Finally, as this is a retrospective description of an intraoperative emergency, certain temporal details and intervention sequences may be subject to recall bias.

## 4. Conclusion

This case underscores the potential for VAE during pediatric bone marrow lavage. There is a critical need for early recognition through ET CO_2_ monitoring, rapid substantiation through echocardiography, and immediate intervention. To prevent severe complications and guarantee safe outcomes during pediatric open bone cavity procedures, it is imperative to be aware, employ meticulous lavage techniques, and be prepared for emergencies.

## Acknowledgments

The authors acknowledge the multidisciplinary team at Kunming Children’s Hospital for their collaborative efforts in managing this emergency.

## Author contributions

**Conceptualization:** Bo Feng.

**Formal analysis:** Hong Duan.

**Investigation:** Hong Duan.

**Methodology:** Kai Liu, Li-ming Cheng.

**Writing – original draft:** Bo Feng.

**Writing – review & editing:** Kai Liu, Li-ming Cheng.
